# Increased Computed Tomography Utilization in the Emergency Department and Its Association with Hospital Admission

**DOI:** 10.5811/westjem.2017.5.34152

**Published:** 2017-07-19

**Authors:** M. Fernanda Bellolio, Herbert C. Heien, Lindsey R. Sangaralingham, Molly M. Jeffery, Ronna L. Campbell, Daniel Cabrera, Nilay D. Shah, Erik P. Hess

**Affiliations:** * Mayo Clinic, Department of Emergency Medicine, Rochester, Minnesota; † Mayo Clinic, Robert D. and Patricia E. Kern Center for the Science of Health Care Delivery, Rochester, Minnesota; ‡ Mayo Clinic, Division of Health Care Policy and Research, Rochester, Minnesota; § OptumLabs, Cambridge, Massachusetts

## Abstract

**Introduction:**

Our goal was to investigate trends in computed tomography (CT) utilization in emergency departments (EDs) and its association with hospitalization.

**Methods:**

We conducted an analysis of an administrative claims database of U.S. privately insured and Medicare Advantage enrollees. We identified ED visits from 2005 through 2013 and assessed for CT use, associated factors, and hospitalization after CT, along with patient demographics. We used both descriptive methods and regression models adjusted for year, age, sex, race, geographic region, and Hwang comorbidity score to explore associations among CT use, year, demographic characteristics, and hospitalization.

**Results:**

We identified 33,144,233 ED visits; 5,901,603 (17.8%) involved CT. Over time, CT use during ED visits increased 59.9%. CT use increased in all age groups but decreased in children since 2010. In propensity-matching analysis, odds of hospitalization increased with age, comorbidities, male sex, and CT use (odds ratio, 2.38). Odds of hospitalization over time decreased more quickly for patients with CT.

**Conclusion:**

CT utilization in the ED has increased significantly from 2005 through 2013. For children, CT use after 2010 decreased, indicating caution about CT use. Male sex, older age, and higher number of comorbidities were predictors of CT in the ED. Over time, odds of hospitalization decreased more quickly for patients with CT.

## INTRODUCTION

Computed tomography (CT) is both screening tool and diagnostic tool, with widespread application for evaluation of numerous conditions and diagnosis of complex medical problems.^1^–^3^ CT utilization has increased in the emergency department (ED) in the United States and Canada^4^ without a corresponding change in diagnostic yield^5^ and with disproportion to growth in ED patient volume.^6^ These findings may suggest that incremental CT use is of lower value.^7^ The availability of CT scanners may have created a supply-induced demand, which may contribute to increased use and variability in practice without a corresponding increase in quality of care.^8^,^9^

A recent study reports that overall utilization rates were stable for all types of CT across a 10-year period;^10^ however, CT use in the ED increased by more than 80% and decreased by nearly 10% in primary care. That CT use has increased in the ED suggests that EDs are becoming diagnostic centers.^10^ The increased use of ED-based imaging may be related to easy access to imaging and radiology services and to expedited care compared with a clinic setting. In the ambulatory setting, imaging use might be decreasing secondary to factors such as implementation of cost-saving strategies and scrutiny of the appropriateness of use.^11^

Several studies have shown variation among ordering patterns of emergency physicians regarding all CT types and a substantial increase in CT use in the pediatric population.^12^ As the technical quality and speed have improved in medical imaging, clinical decisions have relied increasingly on CT and other imaging techniques.^7^ However, the relationship between CT and hospital admission has not been well studied. We aimed to examine trends of CT use in the ED, investigate causes of varied CT utilization, and evaluate the association between CT use and hospital admission among ED patients.

## METHODS

### Study Design and Setting

We assessed administrative claims data from OptumLabs, a database including privately insured and Medicare Advantage enrollees throughout the U S.^13^ The database has longitudinal health information of more than 100 million enrollees of the past 20 years from geographically diverse regions, with the South and Midwest represented the most.^14^ A subset of enrollees has insurance plans that provide full coverage for professional (e.g., physician), facility (e.g., hospital), and outpatient prescription medication services. Medical claims for professional and facility services include *International Classification of Diseases*, *Ninth Revision, Clinical Modification* (*ICD-9-CM*)*,* diagnosis codes; *ICD-9-CM* procedure codes; *Current Procedural Terminology, Fourth Edition*, (CPT-4) procedure codes; Healthcare Common Procedure Coding System procedure codes; site of service codes; and provider specialty codes. We accessed study data using techniques compliant with the Health Insurance Portability and Accountability Act of 1996. Because this study involved analysis of preexisting, de-identified data, it was exempt from institutional review board approval. This study adheres to the Reporting of Studies Conducted Using Observational Routinely Collected Health Data statement (RECORD).^15^

### Selection of Participants

All patients who presented to an ED from 2005 through 2013 were identified. We assessed changes in CT use over time, associated factors, and disposition after CT use among patients of all ages. Patients were required to have six months of continuous enrollment before their index ED visit dates.

### Data Collection

The demographic variables collected were birth year, sex, and race. We grouped age into six categories: <18, 18 to 34, 35 to 49, 50 to 64, 65 to 79, and >79 years. Race was grouped into White, Black, Hispanic, Asian, and “other.” CT procedures were extracted using standardized CPT-4 codes.

Population Health Research Capsule

What do we already know about this issue?

Computed tomography utilization in the ED has increased without a corresponding change in diagnostic yield and with disproportion to growth in ED patient volume.

What was the research question?

Investigate trends in CT utilization in the EDs and its association with hospital admission using administrative claims.

What was the major finding of the study?

CT use increased in all age groups but decreased in children since 2010. Hospitalization was associated with increasing age, comorbidities, male sex, and CT use. Odds of hospitalization over time decreased more quickly for patients with CT.

How does this improve population health?

CT utilization in the ED has increased significantly from 2005 through 2013. For children, CT use after 2010 decreased, indicating caution about CT use. Over time, odds of hospitalization decreased more quickly for patients with CT, suggesting a diagnostic hub role for emergency departments.

We categorized CT into the body regions *head*, *chest*, *abdomen*, and *other*. Abdominal CT included imaging of the abdomen solely and of the abdomen and pelvis. Scans grouped as *other* included various, relatively uncommon CT evaluations of spine, extremities, neck, and sinuses. To decrease the risk of overestimating utilization of CT, we collapsed multiple procedures for the same body region performed on the same day into one CT event. CT performed for hospitalized patients was not included.

The primary diagnosis from each CT scan was taken using diagnosis codes from administrative claims data and with clinical classification software (CCS) created by the Agency for Healthcare Research and Quality (AHRQ) to organize these diagnoses into diagnostic categories. The outcomes of interest for the study were CT performed in the ED and its relationship with hospital admission. Patients admitted under observation status or placed in an observation unit did not count as in-patient stays.

### Statistical Analysis

We calculated utilization rates per 1,000 ED visits across groups defined by baseline characteristics. Overall CT utilization trends were examined by patient age and sex, U.S. region, year, and CT body area. We reported rates of hospital admission of patients who received and did not receive a CT as risk ratios (RR) with 95% confidence intervals (CI).

We also estimated adjusted models by year, age, sex, race, U.S. region, and Hwang comorbidity score and explored associations among CT use, year, patient demographic characteristics, and hospitalization. Main outcomes were presented as adjusted odds ratio (OR) with 95% CI.

### Patient Matching

To control for the effect of baseline differences among patients with and without CT, we used both propensity-score matching and exact matching to create two cohorts of similar people with and without the exposure (CT in the ED). The propensity score is the conditional probability of a patient receiving a particular exposure—in this case, initial CT exposure—given a set of potential confounders. To calculate propensity scores, we included the confounders in a logistic regression model to predict exposure without including outcome.^16^,^17^ Patients with the same propensity score have the same adjusted probability of receiving CT, though some ultimately received a CT while others did not.

The propensity score was estimated using logistic regression. We matched by age, sex, race, number of comorbidities (baseline Hwang comorbidity score), U.S. region, race, year of ED visit, Berenson-Eggers Type of Service indicators and exact match on diagnosis group. To check the balancing properties of the propensity score, we compared standardized differences in patient characteristics before and after propensity-score matching^18^ (Appendix Figure 1).

To ensure that matched patients were being seen in the ED for similar reasons, we determined the Hierarchical Condition Category [HCC] from AHRQ’s CCS for the primary diagnosis for ED visit. This classification system categorizes all *International Classification of Diseases, 9^th^* *Revision, Clinical Modification* (I*CD-9-CM*) diagnosis into a limited number of categories or diagnosis groups. Finally, we further controlled for baseline differences by matching exactly on age, sex, primary diagnosis HCC, and baseline Hwang comorbidity score. As a result, each person who received a CT in the ED is matched to a person of the same age, sex, primary diagnosis HCC, and baseline Hwang score, and with a propensity score for CT use within nearest neighbor with a 1:1 ratio, which additionally accounts for patient race, visit year, types of services received in the ED, and region of the U.S.

We conducted analyses with SAS software version 9.3 (SAS Institute Inc), and Stata version 14 (StataCorp LP). Statistical significance was set at *P* less than 0.05 for modeling.

## RESULTS

### Trends in CT Use over Time

Of the identified 33,144,233 ED visits, 5,901,603 (17.8%) had a CT associated with the visit. Total ED visits increased over time from 3,079,601 in 2005 to 4,324,993 in 2013 (a 40.4% increase). CT use during ED visits increased 59.9%, from 153.0 CTs per 1,000 visits in 2005 to 245.1 per 1,000 in 2013.

Over time, female and male patients underwent CT at similar rates (151.7 and 154.6 per 1,000 ED visits in 2005 vs 245.3 and 244.9 in 2013, respectively) (Table 1). CT use increased in all age groups; the greatest growth occurred in the older population (45.2% increase in patients aged 65 to 79 years and 47.3% increase in those older than 79 years). In the pediatric population, CT exposure peaked in 2010 at 85.2 scans per 1,000 visits and decreased to 72.7 per 1,000 visits in 2013.

**Figure 1:**
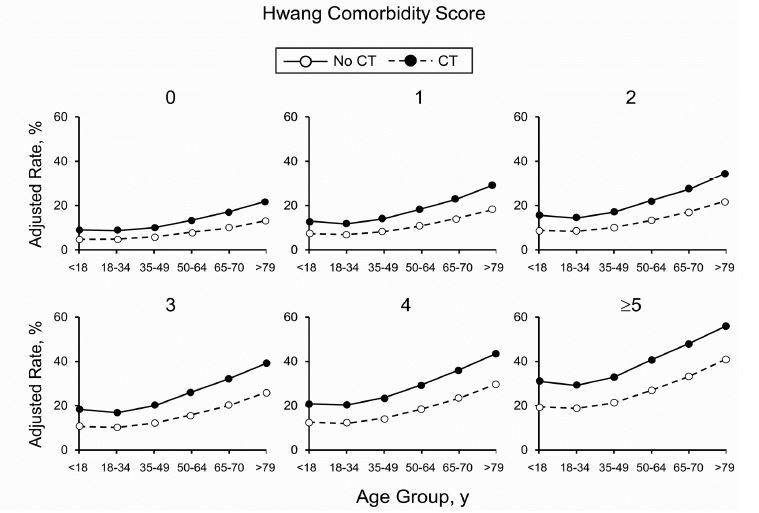
Rates of admission to the hospital by patient comorbidities (Hwang comorbidity score). Age and CT performed in the emergency department among the matched cohort.

Patients with more comorbidities as measured with Hwang comorbidity score had greater increases in CT rates over time, with CT use increasing 36.9% for a 0 score and 45.7% for a score of 5 or higher. Those with Hwang score of 0 had a CT rate of 132.5 compared with 385.3 for those with a Hwang score of 5 or higher (Table 1 and Figure 1).

#### Trends in Hospital Admission

**Figure 2:**
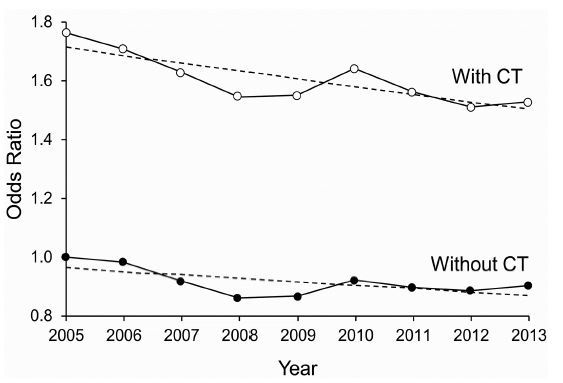
Odds of admission to the hospital associated with CT performed in the emergency department over time.

The rate of hospital admission increased 21.6% in the same period, going from 119.1 per 1,000 ED visits in 2005 to 144.8 per 1,000 ED visits in 2013. Overall, patients who received CT in the ED were more likely to be admitted to the hospital than those who did not receive it in 2005 (unadjusted RR [95% CI], 2.90 [2.88–2.91]) vs 2013 (unadjusted RR [95% CI], 2.29 [2.28–2.30]) (Table 1, Figure 2). Younger patients who had CT in the ED were less likely to be admitted to the hospital, with a 26.52% decrease in hospital admission for patients younger than 18 years and an 8.40% decrease for patients aged 18 to 34 years. CT in the ED was associated with increased admission rates in patients older than 50 years from 2005 to 2013. Male patients were more likely to be admitted to the hospital than female patients (from 123.3 to 154.7 vs 115.6 to 137.0 per 1,000 ED visits in 2005 and 2013, respectively). Patients with a Hwang comorbidity score of 0 or 1 were less likely to be admitted to the hospital after CT.

#### Matched Cohort Trends of CT Use

We performed propensity matching to evaluate the relationship between CT use and hospital admission. In total, 2,119,962 pairs were matched by age group, sex, race, U.S. census region, number of comorbidities, year of ED visit, baseline Hwang comorbidity score, and exact match on diagnosis group (Appendix Table 1). We used standardized differences to evaluate how effectively the propensity score balanced the matched cohorts. All variables were within the 10% threshold, showing that matching achieved balance across the groups (Appendix Figure 1).

Similarly to the trend analyses, the matched cohort analysis found that overall, the rates of hospital admission increased with increasing age for patients older than 50 years (OR, 1.20 for age 50–64 years; 1.74 for 65–79 years; and 2.36 for >79 years), male sex (OR, 1.15), and increasing Hwang comorbidity score (OR, 3.34 for a score of 2; 5.15 for 4; and 7.25 for ≥5) Table 2. Among body areas, CT of the head and abdomen were the most common. CT for all types of body areas has increased over time (Appendix Figure 2).

#### Propensity-Matched Cohort Hospital Admission

Overall CT utilization in the ED increased over time, and the odds of being admitted to the hospital decreased. Among patients with CT, the odds of hospital admission decreased each year of the study (Figure 2), with a 42% decrease from 2005 through 2013. When evaluating the change in OR over time and determining the interaction between CT and year, we found that the rate of change over the years was significantly different for patients who received CT vs. those who did not (*P*<0.001). The odds of admission decreased faster among patients with CT than those without CT. The absolute decrease in the odds of hospital admission was greater among patients who had CT than those who did not.

## DISCUSSION

In this study of CT use trends in the ED, healthcare delivery variation and its association with hospital admission rates, we found that CT during ED visits increased almost 60% from 2005 to 2013. Overall, CT use increased in all age groups and particularly in the oldest population (>79 years). However, a slight decline in CT use was found among the pediatric age group (<18 years) after 2010, perhaps secondary to the widespread adoption of pediatric clinical decision rules.^19^

Patients with CT performed in the ED were more likely to be admitted to the hospital. However, over the nine years, the ratio of admission among those with CT decreased faster than among those without CT during the ED visit, possibly indicating that CT is used both for diagnostic and risk stratification and guides admission decisions.

Patients with a major procedure, endoscopy or dialysis or who needed anesthesia on the date of the ED visit were more likely to have CT and be admitted to the hospital. This outcome probably suggests a strong relationship between disease complexity and CT utilization. This decrease in admission rates may be secondary to the increase in use of observation services and admission under observation status and not to a real decrease in the number of patients hospitalized.^20^,^21^

EDs increasingly support primary care providers through their complex diagnostic work-ups that cannot be performed in physician offices. EDs also augment primary care providers by managing case overflow, after-hours cases, and weekend demand for medical care.^22^ In some cases, CT allows clinicians to avoid a hospital admission by providing the information necessary to make a definitive diagnosis.^23^ By 2010, nearly one-half of ED visits included at least one imaging test,^24^–^29^ influenced by increased fear of malpractice litigation and patients’ expectations.^29^–^35^

The news media, policymakers, patients, and healthcare providers have called CT utilization into question^23^,^36^,^37^ because diagnostic imaging is considered one of the key drivers of increasing healthcare cost in the U.S.^38^ One study reported that use of abdominal CT was associated with decreased revisits,^39^ but other studies have suggested that outcomes are not necessarily improved with more imaging.^40^–^44^ The prevalence of over-testing, over-diagnosing, and over-treating has been criticized in modern medicine. Emergency physicians on a survey reported use of unnecessary testing in EDs, and 97% reported that at least “some” advanced imaging that they personally order is medically unnecessary.^45^

Attempts to reduce the cost of diagnostic imaging procedures in the past decade have been either reducing payments per procedure^46^ or imposing more thoughtful decisions about healthcare delivery, such as the Choosing Wisely initiative.^47^ Analyzing trends in utilization helps healthcare systems understand whether these attempts were successful and identify gaps that should be addressed.

With the aging of the population and the increased use of EDs, the likelihood of CT performed in the ED is increasing. Variations on CT use have been associated with patient characteristics (i.e., age, race, insurance status, sex, and diagnoses)^48^ and associated less with hospital characteristics (e.g., number of beds, hospital teaching status). Understanding variations in CT utilization can help identify underuse and overuse, both of which may be costly and negatively affect healthcare quality.^3^,^48^,^49^

A study by Horný, Burgess, and Cohen^50^ from 2011 to 2013 showed that visits resulting in CT decreased over time, and diagnostic ultrasonography increased at a higher rate than the decrease in CT use. In our cohort of the present study, the pediatric population had a decrease in CT use since 2010. The awareness of providers and patients regarding radiation exposure-induced malignancies may have influenced the decreased CT use, as well as robust and validated decision rules.^19^,^51^–^53^

Imaging increases ED length of stay and poses a risk of misreading the imaging result and incidental findings.^54^–^57^ The latter can lead to increased utilization from downstream testing that may be unnecessary. Morris et al^14^ recently showed that CT coronary angiography in the ED was associated with increased downstream healthcare utilization, repeat testing, hospitalization, return ED visits, and later invasive procedures, such as coronary angiography and stent placement, compared with functional stress testing. This increase in downstream utilization could be due to suboptimal patient selection, unclear physiologic significance of coronary lesions identified on CT, or lack of standardization regarding how to best manage cases on the basis of the degree of coronary stenosis identified.

In the present cohort of privately insured and Medicare Advantage patients, CT utilization increased over the study period. Patients with CT in the ED had decreasing hospital admission rates over time at a higher rate than those without CT. This observation might indicate that CT is able to identify patients who can benefit from inpatient admission, and it appears to be a diagnostic tool to aid in determining appropriate disposition and risk assessment. This finding may be particularly relevant to patients who require major procedures and those with complex clinical presentations (e.g., elderly persons, patients with multiple chronic medical conditions).

## LIMITATIONS

Administrative claims data are susceptible to coding errors, and problems like undercoding comorbidities or miscoding diagnoses are possible. Each individual claim may not include all of a patient’s diagnoses, resulting in underreporting of comorbidities. To mitigate this limitation, we restricted the analysis to patients with at least six months of continuous enrollment before the ED visit, which increases the number of claims on which we base our comorbidity calculation. Second, despite use of propensity matching, there is potentially unmeasured confounding between the groups. In our propensity score, we included all available potential confounders and obtained propensity scores with a standardized difference of less than 0.1 for the covariates. Models that automatically select the variables to calculate the propensity score can reduce bias relative to models that use only a predefined group of variables.^58^ Therefore, we supplemented a defined set of a priori confounders with additional covariates for all medical conditions and demographic characteristics.^59^–^61^

Third, we did not have access to data from uninsured or Medicaid patients. This is a potential source of bias, as it is possible that CT ordering patterns differ in these populations. Fourth, the need for CT and hospital admission might be markers of the severity of the underlying illness. To account for these differences, we adjusted data using the Hwang comorbidity score and matched for ED diagnosis. However, we acknowledge that comorbidities are only part of the severity of illness. We did not evaluate whether CT utilization translated into increased downstream healthcare utilization, including critical care unit use, surgery or procedures, and death.

Another limitation is the possibility that some patients were hospitalized for “observation stays” or placed in an observation unit, and despite occurring in the hospital, observation stays do not count as inpatient stays. This might result in increased rates of outpatient visits with CT use that did not result in hospitalization.

### Future Directions

With the increase in the adoption of electronic health records, there has been an increase in the amount of data available for the study of ED imaging. Multicenter data sets are now available to investigators.^62^ Overuse, underuse, and misuse of healthcare services affect the quality and cost of care. There are estimates that up to one-third of all U.S. healthcare spending produces no benefit to the patient and some results in harm,^63^ with approximately $600 billion of avoidable cost to the healthcare system each year.^42^,^62^

Of paramount importance is assessment of patterns of healthcare utilization and effects on practice, with naturalistic understanding of the clinical behaviors of providers. It appears that CT utilization is driven in part not by a diagnostic goal but by a risk-stratification and disposition goal defined by EDs that function as diagnostic and imaging centers. Implementing evidence-based decision supports and aids to increase the understanding of providers’ behavior (e.g., Pediatric Head CT rule)^19^ are promising approaches for future interventions to decrease CT overuse and radiation exposure, increase practice efficiency, and decrease healthcare costs for patients being considered for CT.

## CONCLUSION

CT utilization in the ED has significantly increased during 2005 through 2013, for which an increasing comorbidity number, male sex, and older age were predictors of CT use. Having CT in the ED increased the odds of hospital admission. Over time, patients who had CT in the ED decreased their admission rates at a faster pace than those without CT, particularly patients with high acuity and complex clinical presentations.

## Supplementary Information







## Figures and Tables

**Table 1:** Trends of CT use in the ED and relationship with hospital admission, 2005–2013.

Patient characteristic	Rate Per 1,000 ED visits by year									% Change over time

	2005 (Reference)	2006	2007	2008	2009	2010	2011	2012	2013	
Hwang comorbidity score, CT obtained										
0	96.79	104.01	111.35	116.88	125.00	131.72	130.80	130.59	132.54	36.93
1	148.37	158.36	168.83	175.38	184.97	194.27	194.62	194.10	198.27	33.63
2	178.91	192.85	205.39	211.80	224.52	235.54	235.67	235.24	242.57	35.58
3	207.20	217.90	234.23	244.53	257.66	268.35	270.41	273.26	281.90	36.06
4	226.71	240.93	260.42	267.92	285.87	296.86	302.36	304.50	317.36	39.98
≥5	264.45	282.87	303.34	317.76	338.74	349.88	358.74	368.35	385.26	45.68
Hwang comorbidity score, CT obtained and patients admitted										
0	14.74	15.49	15.55	15.44	15.28	15.95	15.01	13.95	13.86	−6.00
1	31.76	32.52	32.75	32.12	33.01	34.49	32.58	31.59	30.81	−2.98
2	49.41	51.51	51.92	51.66	52.49	55.34	53.40	50.92	51.88	5.00
3	69.58	70.99	73.02	73.43	74.94	78.99	76.52	73.93	74.79	7.49
4	89.12	92.44	96.12	94.32	99.20	102.91	100.20	98.80	100.58	12.85
≥5	136.86	143.53	148.18	153.59	161.77	167.18	167.23	171.55	174.64	27.60
Sex, CT obtained										
Female	151.65	164.95	180.33	190.61	209.50	225.75	229.39	234.29	245.30	61.75
Male	154.63	167.38	182.31	192.81	209.50	225.68	229.34	234.02	244.86	58.35
Sex, CT obtained and patients admitted										
Female	42.86	46.02	49.46	51.43	57.17	64.04	63.73	64.83	67.29	57.02
Male	47.74	51.60	55.02	57.85	64.10	71.89	71.55	73.39	77.02	61.31
Age, y, CT obtained										
<18	67.95	72.91	77.36	78.96	83.31	85.21	79.96	75.55	72.71	7.00
18–34	135.47	145.95	156.38	162.08	170.89	177.95	176.24	175.43	176.00	29.91
35–49	176.04	189.49	202.69	213.27	229.00	237.75	238.44	238.29	244.74	39.03
50–64	209.93	224.96	241.96	253.68	274.15	283.44	288.72	291.72	302.35	44.03
65–79	251.40	265.12	287.23	300.70	322.81	332.78	342.03	349.34	365.08	45.22
>79	305.43	321.80	351.03	373.29	399.95	409.62	420.07	433.25	450.00	47.33
Age, y, CT obtained and patients admitted										
<18	12.30	12.52	12.38	12.38	11.35	11.90	10.73	9.50	9.04	−26.52
18–34	25.74	26.96	27.18	26.65	27.08	27.41	25.90	23.94	23.58	−8.40
35–49	43.89	46.21	47.24	47.72	49.29	50.60	47.06	44.88	44.08	0.45
50–64	76.06	80.23	83.18	84.40	89.94	92.64	90.79	87.65	88.43	16.27
65–79	124.50	130.88	135.39	141.08	149.25	151.30	150.73	154.09	155.57	24.96
>79	176.13	178.91	186.03	196.83	206.71	209.00	208.72	217.29	217.76	23.64

*CT*, computed tomography; *ED*, emergency department.

**Table 2:** Odds ratios of hospital admission among 2,119,962 patients with and without CT in the propensity-matched cohort

	CT	No CT
		
Characteristic	Odds ratio (95% CI)^a^	Odds ratio (95% CI)^a^
Age, y		
<18	Reference	Reference
18–34	0.94 (0.929–0.962)	0.87 (0.853–0.895)
35–49	1.11 (1.089–1.127)	1.11 (1.083–1.135)
50–64	1.50 (1.473–1.525)	1.52 (1.483–1.554)
65–79	2.30 (2.250–2.344)	2.29 (2.233–2.354)
>79	2.95 (2.890–3.016)	3.26 (3.171–3.345)
Sex		
Female	Reference	Reference
Male	1.19 (1.179–1.197)	1.19 (1.179–1.201)
Race		
White	Reference	Reference
Asian	1.08 (1.055–1.112)	1.10 (1.068–1.139)
Black	1.00 (0.981–1.009)	0.96 (0.942–0.973)
Hispanic	1.05 (1.033–1.063)	1.07 (1.049–1.087)
CCS Group No. on ED visit	1.02 (1.017–1.018)	1.02 (1.017–1.017)
Year of ED visit		
2005	Reference	Reference
2006	0.94 (0.921–0.953)	0.96 (0.939–0.978)
2007	0.85 (0.833–0.862)	0.85 (0.831–0.866)
2008	0.77 (0.759–0.786)	0.76 (0.742–0.774)
2009	0.75 (0.736–0.761)	0.73 (0.715–0.745)
2010	0.73 (0.714–0.739)	0.70 (0.687–0.715)
2011	0.67 (0.657–0.679)	0.65 (0.640–0.666)
2012	0.61 (0.602–0.622)	0.61 (0.603–0.627)
2013	0.58 (0.569–0.587)	0.58 (0.569–0.592)
Hwang comorbidity score		
0	Reference	Reference
1	1.52 (1.504–1.544)	1.58 (1.556–1.612)
2	2.05 (2.017–2.075)	2.25 (2.206–2.289)
3	2.54 (2.506–2.583)	2.89 (2.837–2.950)
4	2.98 (2.933–3.033)	3.48 (3.410–3.555)
^3^5	3.86 (3.801–3.921)	4.61 (4.519–4.701)
BETOS indicators during ED visit		
Anesthesia use	6.54 (6.358–6.732)	6.77 (6.547–6.991)
Major procedure	6.23 (6.036–6.440)	4.31 (4.175–4.455)
Ambulatory visit	1.92 (1.877–1.967)	0.03 (1.802–1.913)
Minor procedure	0.44 (0.433–0.446)	0.33 (0.325–0.338)
Oncology	1.14 (1.006–1.294)	1.12 (0.971–1.292)
Endoscopy	1.25 (1.203–1.305)	1.18 (1.132–1.240)
Dialysis procedure	1.60 (1.367–1.879)	1.24 (1.059–1.452)
Laboratory test	0.69 (0.681–0.694)	0.76 (0.751–0.767)
Other test	1.56 (1.547–1.572)	2.04 (2.021–2.060)
Echocardiography	2.45 (2.426–2.484)	2.69 (2.655–2.725)

*BETOS*, Berenson-Eggers Type of Service; *CCS*, Agency for Healthcare Research and Quality’s clinical classification software; *CT*, computed tomography; ED, emergency department.

a All P<.001.
