# Supplementary material for: Increased Computed Tomography Utilization in the Emergency Department and Its Association with Hospital Admission
Source: West J Emerg Med. 2017 Jul 19;18(5):835–45. doi: 10.5811/westjem.2017.5.34152 (PMC5576619; doi:10.5811/westjem.2017.5.34152)
Supplement: Supplementary file 1 [file wjem-18-835-s001.docx]

**Appendix Table 1.** Comparison of the two cohorts after propensity matching by CT use

|  | **Cohort, No. (%)** | |
| --- | --- | --- |
| **Characteristic** | **CT Used (n=2,119,962)** | **CT Not Used (n=2,119,962)** |
| Age category, y |  |  |
| <18 | 220,906 (10.42) | 220,906 (10.42) |
| 18-34 | 462,932 (21.84) | 462,932 (21.84) |
| 35-49 | 559,578 (26.40) | 559,578 (26.40) |
| 50-64 | 557,239 (26.29) | 557,239 (26.29) |
| 65-79 | 161,827 (7.63) | 161,827 (7.63) |
| >79 | 157,480 (7.43) | 157,480 (7.43) |
| Sex |  |  |
| Female | 1,178,633 (55.60) | 1,178,633 (55.60) |
| Male | 941,329 (44.40) | 941,329 (44.40) |
| Race |  |  |
| White | 1,723,864 (81.32) | 1,742,376 (82.19) |
| Asian | 45,089 (2.13) | 45,543 (2.15) |
| Black | 192,137 (9.06) | 166,862 (7.87) |
| Hispanic | 158,872 (7.49) | 165,181 (7.79) |
| US census region |  |  |
| New England | 80,924 (3.82) | 82,770 (3.90) |
| Mid-Atlantic | 255,721 (12.06) | 255,038 (12.03) |
| South Atlantic | 553,036 (26.09) | 553,194 (26.09) |
| East North Central | 323,814 (15.27) | 321,887 (15.18) |
| East South Central | 101,823 (4.80) | 102,850 (4.85) |
| West North Central | 189,439 (8.94) | 187,093 (8.83) |
| West South Central | 285,186 (13.45) | 287,638 (13.57) |
| Mountain | 176,175 (8.31) | 176,401 (8.32) |
| Pacific | 153,844 (7.26) | 153,091 (7.22) |
| Year of ED visit |  |  |
| 2005 (reference) | 185,482 (8.75) | 183,392 (8.65) |
| 2006 | 202,858 (9.57) | 202,020 (9.53) |
| 2007 | 209,639 (9.89) | 209,413 (9.88) |
| 2008 | 207,465 (9.79) | 207,458 (9.79) |
| 2009 | 226,586 (10.69) | 226,584 (10.69) |
| 2010 | 228,433 (10.78) | 229,518 (10.83) |
| 2011 | 250,239 (11.80) | 252,519 (11.91) |
| 2012 | 287,570 (13.56) | 288,205 (13.59) |
| 2013 | 321,690 (15.17) | 320,853 (15.13) |
| Hwang comorbidity score |  |  |
| 0 | 623,701 (29.42) | 623,701 (29.42) |
| 1 | 438,406 (20.68) | 438,406 (20.68) |
| 2 | 310,819 (14.66) | 310,819 (14.66) |
| 3 | 231,617 (10.93) | 231,617 (10.93) |
| 4 | 165,670 (7.81) | 165,670 (7.81) |
| ≥5 | 349,749 (16.50) | 349,749 (16.50) |
| Anesthesia use |  |  |
| No | 2,079,646 (98.10) | 2,074,057 (97.83) |
| Yes | 40,316 (1.90) | 45,905 (2.17) |
| Major procedure |  |  |
| No | 2,092,760 (98.72) | 2,088,715 (98.53) |
| Yes | 27,202 (1.28) | 31,247 (1.47) |
| Eye procedure |  |  |
| No | 2,119,189 (99.96) | 2,119,080 (99.96) |
| Yes | 773 (0.04) | 882 (0.04) |
| Ambulatory visit |  |  |
| No | 2,069,788 (97.63) | 2,059,575 (97.15) |
| Yes | 50,174 (2.37) | 60,387 (2.85) |
| Minor procedure |  |  |
| No | 1,935,208 (91.29) | 1,929,155 (91.00) |
| Yes | 184,754 (8.71) | 190,807 (9.00) |
| Oncology |  |  |
| No | 2,118,705 (99.94) | 2,118,603 (99.94) |
| Yes | 1,257 (0.06) | 1,359 (0.06) |
| Endoscopy |  |  |
| No | 2,105,814 (99.33) | 2,104,980 (99.29) |
| Yes | 14,148 (0.67) | 14,982 (0.71) |
| Dialysis procedure |  |  |
| No | 2,119,137 (99.96) | 2,119,119 (99.96) |
| Yes | 825 (0.04) | 843 (0.04) |
| Laboratory test |  |  |
| No | 1,578,484 (74.46) | 1,549,744 (73.10) |
| Yes | 541,478 (25.54) | 570,218 (26.90) |
| Other test |  |  |
| No | 1,470,517 (69.37) | 1,445,770 (68.20) |
| Yes | 649,445 (30.63) | 674,192 (31.80) |
| Echocardiography |  |  |
| No | 1,930,564 (91.07) | 1,938,841 (91.46) |
| Yes | 189,398 (8.93) | 181,121 (8.54) |
