# Supplementary material for: Increased Computed Tomography Utilization in the Emergency Department and Its Association with Hospital Admission
Source: West J Emerg Med. 2017 Jul 19;18(5):835–45. doi: 10.5811/westjem.2017.5.34152 (PMC5576619; doi:10.5811/westjem.2017.5.34152)
Supplement: Supplementary file 2 [file wjem-18-835-s002.docx]

**

 Appendix Figure 1.** Standardized differences pre- and post-match ,using propensity matching to compare differences in characteristics of patients who did or did not receive CT.

*BETOS*, Berenson-Eggers Type of Service; *CCS*, Agency for Healthcare Research and Quality’s clinical classification software; *CT*, computed tomography; *ED*, emergency department *SNF*, skilled nursing facility
