# Supplementary material for: Increased Computed Tomography Utilization in the Emergency Department and Its Association with Hospital Admission
Source: West J Emerg Med. 2017 Jul 19;18(5):835–45. doi: 10.5811/westjem.2017.5.34152 (PMC5576619; doi:10.5811/westjem.2017.5.34152)
Supplement: Supplementary file 3 [file wjem-18-835-s003.docx]

**Appendix Figure 2.** Increase in CT over time by body region and per 1,000 emergency department visits.








Footnote: A, Overall increase in CT use. B, Increase in CT use among patients admitted to the hospital.

*CT*, computed tomography
